# Cyberbiosecurity Challenges of Pathogen Genome Databases

**DOI:** 10.3389/fbioe.2019.00106

**Published:** 2019-05-15

**Authors:** Boris A. Vinatzer, Lenwood S. Heath, Hussain M. J. Almohri, Michael J. Stulberg, Christopher Lowe, Song Li

**Affiliations:** ^1^School of Plant and Environmental Sciences, College of Agriculture and Life Sciences, Virginia Polytechnic Institute and State University, Blacksburg, VA, United States; ^2^Department of Computer Science, Virginia Polytechnic Institute and State University, Blacksburg, VA, United States; ^3^Department of Computer Science, Kuwait University, Kuwait City, Kuwait; ^4^Animal and Plant Health Inspection Service (USDA), Riverdale Park, MD, United States; ^5^Beltsville Agricultural Research Center, Agricultural Research Service (USDA), Beltsville, MD, United States

**Keywords:** cyberbiosecurity, cybersecurity, genome databases, pathogen, plant and animal health

## Abstract

Pathogen detection, identification, and tracking is shifting from non-molecular methods, DNA fingerprinting methods, and single gene methods to methods relying on whole genomes. Viral Ebola and influenza genome data are being used for real-time tracking, while food-borne bacterial pathogen outbreaks and hospital outbreaks are investigated using whole genomes in the UK, Canada, the USA and the other countries. Also, plant pathogen genomes are starting to be used to investigate plant disease epidemics such as the wheat blast outbreak in Bangladesh. While these genome-based approaches provide never-seen advantages over all previous approaches with regard to public health and biosecurity, they also come with new vulnerabilities and risks with regard to cybersecurity. The more we rely on genome databases, the more likely these databases will become targets for cyber-attacks to interfere with public health and biosecurity systems by compromising their integrity, taking them hostage, or manipulating the data they contain. Also, while there is the potential to collect pathogen genomic data from infected individuals or agricultural and food products during disease outbreaks to improve disease modeling and forecast, how to protect the privacy of individuals, growers, and retailers is another major cyberbiosecurity challenge. As data become linkable to other data sources, individuals and groups become identifiable and potential malicious activities targeting those identified become feasible. Here, we define a number of potential cybersecurity weaknesses in today's pathogen genome databases to raise awareness, and we provide potential solutions to strengthen cyberbiosecurity during the development of the next generation of pathogen genome databases.

## 1. Introduction

Current biological research, including pathogen related research projects, are increasingly dependent on public genome databases. Genome databases provide information about genomic sequences (Benson et al., 2018), gene annotations (Aken et al., 2016), protein sequences (Punta et al., 2012), protein interactions, and metabolic networks, which are playing crucial roles in designing and implementing biological experiments in many organisms.

A few key online databases provide repositories of raw data, processed data, and metadata generated by genome-scale sequencing projects (Leinonen et al., 2011a,b). Many specialized databases, such as pathogen-related databases (Winnenburg, 2006; Aurrecoechea et al., 2017; Wattam et al., 2017), provide curated data that serve specific research domains. In the 2018 Nucleic Acids Research (NAR) database issue, an online molecular biology database collection, 1,737 databases were reported as being publicly available. This article will review cybersecurity aspects of online genome databases with a focus on pathogen-related databases. Among the databases collected by NAR, 30 are dedicated to viral genomes, 71 to prokaryotic genomes, and 35 to fungal genomes. These databases are of great interest to pathogen research. Many general-purpose databases also contain data related to pathogen genomes, genes, and protein annotations (Mukherjee et al., 2017). Some online databases not only provide repositories of research data but also provide computational tools that allow users to perform genomic data analysis online (Wattam et al., 2017). As metagenome and transcriptome sequencing become common practice in pathogen research, online databases are important tools for annotating and interpreting these genome-scale experiments.

There has been an increasing number of high-profile cybersecurity breaches in recent years that have raised public awareness of potential social, political, and economic consequences that can be caused by such attacks (Newman, 2018). For example, private health record systems at hospitals have been targets for ransomware attacks in recent years (Osborne, 2018). However, cybersecurity awareness is still lacking in the research and health care industries (Kruse et al., 2017). Despite the importance of online genome databases to biological and pathogenic research, there is limited discussion, and virtually no research that focuses on biosecurity and cybersecurity risks (“cyberbiosecurity”) with regard to online biological databases. We suspect that one reason is that genome databases are most utilized by the research community. The number of people that can be directly affected by cyberattacks on genome databases is currently relatively small as compared to web sites or databases for large enterprises that have millions of users. Because of this perceived limited utilization of genomic data, there is limited incentive to target genome databases. However, given the millions of research dollars that are invested in generating genomic data yearly, it is surprising to see that there is almost no research that has been published related to protecting such data from cyberattacks.

Analogous to the tens of thousands of public libraries that hold the knowledge of humanity in the format of text books, public genome databases hold the entire body of genome research knowledge gained in the past thirty years. The size of public genomic data may someday surpass the size of all published text books combined. Besides the importance of protecting the products of public research investment, cyberbiosecurity research on genome databases is even more important because these databases contain so much of the knowledge gained over many years by the world-wide research community and because of the impact of this knowledge on human, animal, and plant health. Public genome databases also provide a unique resource for cyberbiosecurity research that aims to protect the bioeconomy (Murch et al., 2018; Peccoud et al., 2018), which has been estimated to consist in the USA of as much as 25% of GDP. The cyberinfrastructure and cybersecurity measures for the major biocompanies in health care, biopharma, and in the ag domains are largely unknown to the research community and thus cannot be easily analyzed. Unlike biocompanies, public genome databases have the intention to broaden their impacts by granting and facilitating open access to all users. Additionally, major innovations in computational methods for genomic data analysis are also largely driven by public research and open source software. Many companies, academic institutions, and government entities are likely also using open source software and databases developed in the public research community because the latest innovations in genomics are typically coming from academic research. Therefore, public genome databases provide a front-end of a highly innovative research community and are an ideal data resource for analyzing potential risks for cyberbiosecurity.

In the coming decades, we expect that genomic data will become more widely accessed and contextualized, and thus becoming increasingly relevant to public health and safety. For example, metagenomic sequencing can now be used to trace foodborne pathogen outbreaks (Huang et al., 2017; Kim et al., 2018), which potentially affect tens of thousands of consumers. Metagenomic sequencing is also used in detecting bacterial pathogens (Pendleton et al., 2017; Lazarevic et al., 2018), fungal pathogens (Tong et al., 2017), and viruses (Greninger et al., 2017; Lewandowska et al., 2017) in hospitals. Metagenomic sequencing is used in plant disease detection as well, such as detecting pathogens in wheat (Yiheng Hu, 2019) and other crops (Chalupowicz et al., 2019). For all these applications, reliable and accurate genome databases are essential for correct identification of disease-causing pathogens. A recent study has also shown that metagenomic sequencing can even reveal personal identity (Franzosa et al., 2015). Therefore, similar to the security and privacy concerns for personal genomic information (McGuire et al., 2008), personal metagenomic data is another area where cybersecurity is important in protecting sensitive, private, and health-related genomic data (Zmora et al., 2016). In this work, we present an overview of online pathogen genome databases (section 2), identify a number of potential cybersecurity weaknesses in today's genome databases to raise awareness (section 3), and provide potential solutions to strengthen cybersecurity during the development of the next generation of genome databases (section 4). We focus on pathogen-related databases because of the direct health and agricultural implications of genomic studies of pathogens.

## 2. Online Databases for Pathogen Genome Research

Here, we provide an overview of existing databases that are related to pathogen genome research. We will explain the type of data that are hosted in these public genome databases, the potential usage of these data, and what will be the consequences if these databases are affected by cybersecurity breaches. We will review what types of access users can have to these databases and what the mechanisms are for users to contribute data. Finally, we will try to understand what cybersecurity measures will be needed to ensure the privacy, integrity, confidentiality, and availability of existing pathogen genome databases.

### 2.1. General Purpose Genome Databases With Pathogen Information

Most, if not all, molecular sequence data are deposited in the two major genomic data repositories: genome databases (Benson, 2004) hosted by the National Center for Biotechnology Information (NCBI) at the National Institutes of Health in the United States and genome databases (Hubbard et al., 2002) hosted at the European Molecular Biology Laboratory (EMBL). NCBI and EMBL provide databases for nucleotide sequences, protein sequences, genome assemblies, and genome annotations. Both databases also provide computational tools for users to query these databases through web-based interfaces or through programmatic access, such as using the command line or a programming language to access data stored in these databases. Here we focus on the resources and tools that are most relevant to pathogen genome research.

### 2.2. Sequence Databases at NCBI

The NCBI Assembly database (Kitts et al., 2016) is a database for assembled genomes of different organisms. This database hosts completed assemblies, contigs, scaffolds, and chromosomes. The database currently contains 4,055 fungal, 180,914 bacterial, and 23,816 viral genome assemblies (December, 2018). For each assembly, a summary page provides metadata and connections to other NCBI resources, such as its taxonomy browser (Federhen, 2012), original data in BioSample and BioProject (Barrett et al., 2012), and whole genome sequencing databases. Each assembly also includes detailed information regarding the identity of the data contributor. Both BioSample and BioProject are metadata repositories that save submitter-supplied metadata related to the nucleotide sequences and other data deposited at NCBI. More specifically, each BioSample typically includes descriptions of specific biomaterials, such as the name of a particular strain of bacteria. BioProjects are descriptions of larger projects, which consist of many BioSamples. Sequence data in NCBI are also organized under gene, EST, genome, nucleotide, and protein databases. RefSeq (O'Leary et al., 2016) is a database of annotated genes and genomes including many pathogens. Unlike other databases mentioned above, RefSeq provides genome annotations, so it is more useful for finding functional information for different pathogens. SRA (Leinonen et al., 2011b) is another NCBI database that hosts data from short read and long read sequencing projects. Data stored in the SRA database require extensive computational processing and analysis to convert them to useful whole genome sequences or transcriptome sequences that are more biologically meaningful. GEO data sets and GEO profiles (Clough and Barrett, 2016) are databases for gene expression and genome scale data related to gene regulations. These data were generated for various organisms using different technologies including RNA-seq, microarray, ChIP-seq, and other genomic experiments. GEO databases typically include both metadata and raw data from gene expression analysis. In summary, NCBI hosts dozens of databases that can be roughly characterized as the following: (1) sequence repositories, which include databases such as assembly, genome, gene, EST, nucleotide, and protein; (2) the RefSeq database that provides annotated sequences; (3) BioSample and BioProject databases that provide metadata for data sets deposited in the NCBI databases; (4) SRA that provides a repository of raw sequences requiring further processing to generate actual biologically meaningful data sets; and (5) the GEO database that provides genomic data sets related to regulation of gene expression.

Querying the NCBI databases is the most common usage in genomic pathogen studies. To search data from the NCBI database, a unified web interface is provided that allows querying all databases by any anonymous user. NCBI also allows a user to compose and use URLs to directly retrieve data from some databases. Many data sets in NCBI can also be downloaded anonymously using its FTP server. Programmable access is allowed through software developed by NCBI, including Entrez Programming Utilities (E-utilities) and the SRA-toolkit. E-utilities is a set of software tools that allows users to query the NCBI databases from a command line interface. A user is recommended to perform no more than 3 queries per second, otherwise the IP address of the user will be blocked. If the user's IP is blocked, the user must register through NCBI by providing additional information such as their email address and the tool name that the user is to develop. The reason for requiring a tool name is that some users (bioinformaticians) are interested in developing batch query tools for NCBI data. Starting in December 2018, an API (Application Programming Interface) key is required to perform more than 3 queries per second using E-utilities. An API key can be obtained by registered users of NCBI and is associated with a unique user account. Besides E-utilities, NCBI provides BLAST, which allows the user to query its sequence databases using nucleotide or protein sequences as inputs. SRA-toolkit is a utility software designed for downloading data from the NCBI-SRA database. This is because the data sets deposited in the SRA database are typically much larger than most other types of data sets in NCBI databases. No registration is required to use SRA-toolkit to download data, and there are no clear instructions on whether IP addresses will be blocked for using SRA toolkit if a certain bandwidth is exceeded. Queries sent to a database present a minimal risk to the stored data; however, vulnerabilities in the host system or database interpreter are subject to exploitation when vulnerabilities are discovered in the query interpreter. Typically, systems are targeted for attack in order to escalate operating privileges on the host itself, so that its resources can be redirected for the attackers purpose to become a platform for attacks on other systems, or to monetize computing resources by generating digital coinage.

Submitting data to the NCBI database is a multi-step, well-controlled process. For example, to submit a genome assembly to the Assembly database, detailed instructions are provided on the NCBI web site. First, the user is required to login to the BioProject portal using preregistered credentials to establish a project ID and fill out a submission template file. After obtaining the project ID, the user needs to organize the data and metadata of the project into specific formats, such as FASTA and AGP formats. Some data files have to be converted to a specific format using software utilities developed by NCBI.

Once the data files are in the correct format, the user will use another web portal from NCBI to submit and upload the data. After submission, the user will have to send an email to an administrator account at NCBI that includes the description of the project. The process of submitting data to the SRA database is similar to the above described procedures. Because the files that are uploaded to SRA are typically much larger than other data types, the user can use FTP and Aspera Connect to upload files to a predefined FTP folder provided by NCBI administrators by email.

### 2.3. Sequence Databases at EMBL

Genome databases managed by EMBL are mainly through the European Bioinformatics Institute (EBI). Similar to GeneBank (Benson et al., 2018) at NCBI, the European Nucleotide Archive (ENA) (Leinonen et al., 2011a) is a repository of sequence information, including those of pathogens. Some data types are the same between ENA and NCBI, such as assembly and EST data sets. However, these data sets have different sequence identifiers in EMBL compared to NCBI databases. Some data types are similar between ENA and NCBI. For example, ENA uses Sample and Study in place of BioSample and BioProject in NCBI. Some data types are unique to ENA, such as CDS data sets that are found in ENA only. Another major genome database from EMBL is the EnsemblGenomes database (Hubbard et al., 2002). There are multiple databases such as Ensembl Bacteria and Ensembl Fungi that are most relevant to pathogen research. Ensembl Bacteria includes genomes of 44,048 bacterial species, and Ensembl Fungi includes genomes of 811 fungal species. Both databases provide gene and genome annotations. Transcriptome data are available from multiple databases in EBI. ArrayExpress (Kolesnikov et al., 2015) is a database that contains gene expression data and results from other functional genomic assays. Although the name ArrayExpress suggests that the database contains data generated by microarray analysis, the database actually contains RNA-seq, DNA-seq, ChIP-seq, and methylation data, which makes this database very similar to the GEO database in NCBI. Expression Atlas (Papatheodorou et al., 2018) is a curated database for gene expression data only. In regard to raw data of sequencing experiments, SRA also has its counterpart in EMBL, which is also called SRA but is part of the ENA database.

To query data from EMBL databases, a number of methods are available. All databases (ENA, Ensembl genomes, ArrayExpress and Expression Atlas) support text-based queries. ENA also allows sequence-based searches. Several databases provide programmatic access through the REST interface, which allows the user to retrieve data using a URL following a specific syntax. A user can also perform sequence-based searches using REST and SOAP APIs (Application Programming Interface, a commonly shared set of procedures for accessing data across different software platforms). There is a limit of 30 queries at a time if a user uses REST or SOAP to access data. ArrayExpress, ENA, Ensembl Genome, and Expression Atlas all provide FTP access to users for bulk download purposes. Some databases provide additional options for data download. For example, Ensembl Genome databases allow the user to download data by downloading a MySQL dump through their FTP site. Ensembl genome databases also allow users to directly access the MySQL database server with a MySQL client, or by using a PERL API to access a MySQL database. Finally, Biomart (Smedley et al., 2009) is another interface for data access to EMBL databases. A user can use a web interface to interact with Biomart to retrieve data from EMBL databases. Alternatively, a user can use REST, MySQL, a PERL API, or an R API to access data from Biomart.

To submit data to any of the EMBL sequence databases requires processes that are similar to submitting data to NCBI. For example, if a user wants to submit a data set to the ArrayExpress database, the user has to first register an account associated with a user-provided email address and password. The user has to prepare metadata, raw data, and processed data according to a specific format, as required by ArrayExpress. A web interface called Annotare provides detailed, step-by-step instructions on how to upload data through the Annotare web interface.

In this section, we reviewed two of the largest molecular databases in the world, found at NCBI and EMBL. There are several properties that these two databases have in common. Both databases host terabytes of genomic data in many specific data formats. Types of data include metadata, raw data, and processed data. Some data are in text files with specific structures. For example, biological sequences are stored in FASTA and FASTQ format, which are specifically designed for storing molecular sequences, and the correctness of these formats can be checked automatically by computer programs. Some data are binary data, such as SRA data, which require software tools to extract information into human-readable formats. Users can access data using a multitude of methods, including web interface, PERL or R API, MySQL query, REST URL, SOAP, and FTP download. Certain download limits are implemented in both databases to limit the amount of data or the speed of data download by users. To submit data to these databases, a user will need to preregister with an email address and login to use a site-specific web interface to upload data. Metadata, raw data, and processed data can be uploaded, and web forms will need to be filled out to describe the data. Although not explicitly stated in the guideline of the submission process, for both web sites, there are curators that control the final process of integrating user-submitted data into the database.

### 2.4. JGI Genome Databases

The integrated genome and metagenome comparative data analysis system (IMG/M) (Chen et al., 2017) is a database containing tools to annotate microbial genomes and metagenomes. MycoCosm (Grigoriev et al., 2014) is a web portal that hosts fungal genome data. Genomes OnLine Database (GOLD) (Mukherjee et al., 2017) is a database that manages metadata and raw data for genome and metagenome sequencing projects. IMG/M, MycoCosm, and GOLD are all developed by the Joint Genome Institute (JGI) and are supported by the Department of Energy. A large fraction of data in these databases are imported from NCBI Gene Bank and other related databases discussed above. The three JGI databases are unique, because a substantial portion of their genome data are generated by JGI itself. These databases also provide a repository for metagenome sequencing projects, computational tools for gene and genome annotation, and comparative genome analysis. These features are important for functional analysis of microbial genomes but are not clearly present in the NCBI databases or EMBL-EBI genome databases.

### 2.5. Other Specialized Microbial and Pathogen Databases

In addition to the major sequence repositories described above, there are many databases and web services that are of smaller scale and have more specific focus on certain aspects of pathogen genomics. We introduce some of these databases as examples to discuss potential cybersecurity concerns of these databases.

The Pathosystems Resource Integration Center (PATRIC) is a bacterial bioinformatics center (Wattam et al., 2017) that was first established by the National Institute of Allergy and Infectious Diseases (NIAID) as the National Microbial Pathogen Data Resource (NMPDR) (NMPDR, 2019). The major focus of PATRIC is bacterial genome annotation and analysis. There are 202,602 bacterial genomes hosted at PATRIC currently; however, there are also thousands of archaea and phage genomes available in PATRIC. PATRIC provides users with resources in genome, transcriptome, protein interaction, protein structure, signaling pathways, and metabolic pathways annotations for these genomes. Metadata annotation for these data resources are also available. PATRIC also provides analytic tools that allow any user to perform genome assembly, genome annotation, proteome comparisons, RNA-seq analysis, variant analysis, and metabolic pathway model reconstruction. PATRIC also allows users to upload their own gene expression data to perform analysis online. To use these services at PATRIC, a user needs to provide an email address, which will receive a link to a page that allows users to set up a password. Once logged in, the user can use a web-based interface to define their analytic pipelines using a number of published software packages.

PATRIC represents a very common model of genome databases. First, the PATRIC database collects data from multiple external resources including NCBI GeneBank, genome sequencing centers, and other collaborators. Second, a unified pipeline was developed to provide annotation to the sequence data and the processed results are stored in the PATRIC database. Metadata is curated by the PATRIC team and also deposited in the PATRIC database. Third, an external user can use computational tools and computing power provided by PATRIC to analyze user-generated data. To incorporate new user data into the PATRIC database, the user has to contact the PATRIC team through email, and the data will be curated by the PATRIC team before integration into the PATRIC database, although there are no defined industry standards for the curation activities.

There are many additional pathogen databases that are available, and here we provide a brief survey. The Eukaryotic Pathogen Genomics Database resource (EuPathDB) is a collection of databases for Eukaryotic pathogens, their related, non-pathogenic species, and selected host genomes (Aurrecoechea et al., 2017). EuPathDB provides genome, gene, protein, and metabolic pathway annotation as well as many other resources. EuPathDB also provides curated phenotypes, copy number variation, and polysomal transcriptomic data. EuPathDB allows users to build their analysis pipeline through a Galaxy workspace and some user-defined pipeline that can be made public. ViPR is a virus pathogen database (Pickett et al., 2012), which provides a web interface to search genome sequences, gene sequences, protein sequences, and protein structures. Online tools are provided for phylogenetic analysis, comparative genomic analysis, and genome annotation. PHI-base (Winnenburg, 2006; Urban et al., 2017) is a curated database for genes related to host-pathogen interactions. Currently, PHI-base contains information regarding 6438 genes and 11340 interactions between 263 pathogens and 194 hosts. PHI-base includes pathogen information for animal, plant, and fungal pathogens. A user must register before downloading data from PHI-base; however, searching the database does not require registration. PHIDIAS (Xiang et al., 2007) is a curated online database focused on genome, protein domain, and gene expression data related to pathogen and host interactions. Victors (Sayers et al., 2018) is a newly published system under the PHIDIAS database. The focus of Victors is on virulence factors and, currently, there are 5,296 virulence factors stored in the Victors database.

For plant pathogen related resources, PAMDB is a database and website for Plant-Associated Microbes (Almeida et al., 2010) and is designed to store and search data for multi-locus sequence typing for plant pathogenic bacteria. PhytoPath (Pedro et al., 2016) is an online database for genome data of plant pathogens. PhytoPath integrates a genome browser from Ensembl genomes and also provides links to PHI-base.

GenomeTrakr (Allard, 2016) is an FDA-led network of open source, whole genome sequencing projects that involves state, federal, international, and commercial partners. The goal of the GenomeTrakr project is to track food-borne pathogens through whole genome sequencing. One unique feature of the GenomeTrakr project is that there is no centralized data repository for this project hosted by FDA. Data generated from the GenomeTrakr project are deposited under the NCBI BioProject and SRA databases. Database for Reference Grade Microbial Sequences (FDA-ARGOS) is another FDA-led project that has generated high quality, reference-grade genomes for 2000 biothreat microorganisms and common clinical pathogens. The results of this project are also deposited as BioProjects in an NCBI database. These genome databases are summarized in Table 1.

**Table 1:** Functions of genomic databases.

**Functions of online databases**	**Names of online databases**
A. Contain genome, transcriptome, proteome sequences.	NCBI, EBI, DDBJ, JGI, PATRIC, EuPathDB, PAMDB, PHI-base, PHIDIAS, ViPR
B. Contain genome, transcriptome, proteome annotation.	NCBI, EBI, DDBJ, JGI, PATRIC, EuPathDB, PAMDB, PHI-base, PHIDIAS, ViPR
C. Provide raw data repository.	NCBI, EBI, DDBJ, JGI
D. Provide processed data.	NCBI, EBI, DDBJ, JGI, PATRIC, EuPathDB, PAMDB, PHI-base, PHIDIAS, ViPR
E. Include metadata.	NCBI, EBI, DDBJ, JGI, PATRIC, EuPathDB, PAMDB, PHI-base, PHIDIAS, ViPR
F. Include single purpose bioinformatics tools such as BLAST as a service or query tool.	NCBI, EBI, DDBJ, JGI, PATRIC, EuPathDB, PAMDB, PHI-base, PHIDIAS, ViPR
G. Include analysis pipeline build.	PATRIC, EuPathDB, ViPR
H. Upload data access control.	NCBI, EBI, DDBJ, JGI, PATRIC, EuPathDB, PAMDB, PHI-base, ViPR
I. Complete download data access control.	JGI, PAMDB
J. Require strong password.	None
K. Allow programmatic access.	NCBI, EBI, DDBJ, JGI (Globus), PATRIC, EuPathDB

As can be seen in this summary table, all databases described in this review provide metadata (Table 1, E) associated with sequence data (Table 1, A) and sequence data annotation (Table 1, B, D). Inclusion of standardized metadata in genome databases to facilitate data interpretation and data reuse has been a major focus of the genomic research community in the last two decades (Brazma et al., 2001; Brazma, 2009). Because of the awareness of the importance of metadata, including meta-data has become a standard for current genome databases. We also found that only four major databases contain raw data repositories and some raw data can only be found in a single raw data repository. This is because maintaining large amounts of raw sequence data is cost-prohibitive for smaller institutions. However, the current situation does introduce a high risk of data loss in the event that one of these raw data repositories is disrupted and redundancy measures fail, resulting in substantial data loss.

Most databases allow the use of bioinformatics tools such as BLAST, which is used to perform similarity-based queries of a genome database with user-provided input sequences (Table 1, F). There are a limited number of such tools that are available and most of these tools are open source and have been widely used. Although security issues with these tools have not been reported, even if such security risk were to exist, it is relatively easy to control by dedicated measures. For example, funding could be provided to qualified individuals or entities to routinely check the security risk of these few, widely used computational tools. However, what concerns us more is that there is a growing number of a new generation of databases, which provide the users with the capacity to build customized analytical pipelines, composed of distributed compute and storage resources across multiple physical and virtual systems of unknown integrity. Unlike BLAST, some computational tools used in these customized pipelines may not be widely scrutinized. As the genomic data analytics community continuous to grow, more highly specialized new tools are likely to emerge. Data processing pipelines composed of many newly developed computational tools are more susceptible to contain intentional or unintentional vulnerable code or shared libraries and may be much more difficult to maintain and may become more difficult to mitigate security risks.

Another important feature of these databases is that all databases require access control when users request data upload to the main database (Table 1, H). There is always “a human in the loop” to curate and authenticate the user before data are integrated into the database, although there also could still be risk associated with large data uploads when a complete sanity check is computationally prohibitive (see next section). In contrast to upload control, one concern is that only two databases ask for complete data access control (Table 1, I). Complete access control means that a user must register and then login before downloading any data from a database. Most databases provide anonymous download without any control, while some databases (such as NCBI and EBI) do provide throttle mechanisms to curb rapid download of multiple records. Finally, the most concerning problem is that none of the databases reviewed here requires strong passwords, which may lead to multiple cybersecurity risks (see next section).

Finally, some of the databases provide methods for programmatic access, which is to help the users to perform structured queries with programming languages (PERL API) or relational databases (SQL query), or provide faster download speed with external services or fast downloading protocols such as globus and ascp. The risk of using these third party software tools is related to each individual software and can be mitigated accordingly. Since many of these tools are broadly used outside the genomic research community, a simple way to mitigate risk is to raise awareness of genomic research programmers and database managers in the security risk announcements for these computational tools.

## 3. Security Threats

Cybersecurity broadly focuses on the confidentiality, integrity, and availability of digital information (Jang-Jaccard and Nepal, 2014) of all types, including genomic data. Yet there has not been a systematic study concerning security breaches of genome databases. However, personal medical information subjected to ransomware attacks has been reported (Kruse et al., 2017). This topic is not within the scope of this review. Although there is no public report for security breaches of molecular databases, existing cyberattack methods could easily target current molecular databases. We discuss the potential damages that can be caused by cyberattacks to genome databases as summarized in Table 2.

**Table 2:** General security threats for genome databases.

**Threat**	**Impact**	**Remedy**
Confidentiality	Privacy of individuals, leaking credentials	Encryption, strong authentication, access control, data anonymization
Data Integrity	Invalid data	Strong identity verification (such as the use of certificates), encryption, checksum verification
Data Availability	Query performance, denial of service	Distributed data providers, intrusion detection and prevention

**Confidentiality**. One major motivation behind cyberattacks is to gain access to sensitive personal information. Most public genome databases do not contain sensitive personal information such as credit card numbers or social security numbers, yet they do contain individual's genomic data, perhaps the most “personal” data of all. Two reasons that genome databases have not been targeted by cyberattacks is that (1) the population of users of genome databases are mainly research scientists, and accounts for a small percentage of the entire population; and (2) the technology needed to exploit the data has been sophisticated and expensive. However, growth in the field has led to both of the factors eroding in impact. As knowledge and training spreads, and with technological advances, the equipment becomes less expensive and easier to use. Additionally, indiscriminate attacks can always happen and can cause damage to genome databases. A common vulnerability found in this review is that while many databases do require user email and password to establish access control, users repeat their email and password combinations. Thus, credentials compromised in one system could be of interest to attackers to gain access to other accounts of the same user elsewhere. Among the databases we have reviewed, almost no database requires strong passwords, i.e., mandating a sufficiently long password of sufficient complexity (that includes capital letters, numbers, and symbols) to make brute force account password attacks impractical.

A general approach to data confidentiality is to secure the database using methods to maintain data privacy (Bajaj and Sion, 2014). A method with a growing interest is to use encrypted databases (Ravan et al., 2013) with proper access control and high assurance encryption standard. Protecting against privacy attacks, existing methods such as *k*-anonymity (Samarati and Sweeney, 1998; Zhong et al., 2005) can be utilized. Data anonymization can be a challenging task and depends on the structure of the data. Note that methods for data de-anonymization have been suggested (Narayanan and Shmatikov, 2008).

Another major issue with genome databases is using the idea of correlation attacks (Meier and Staffelbach, 1989). The attacker wishes to correlate biological data to specific users or groups of users. The threat can be from authenticated and/or unauthenticated malicious clients. In the first case, an authenticated client is one that has access to the database and can read and correlate records in multiple databases. This is typically referred to as an insider threat and requires a vigilant user review and monitoring process to identify potential candidates. In the second case, the attacker uses a classical external attack, for example exploiting an existing user's credentials, or sending emails to known system users with malware embedded (also known as “phishing”) to gain access to system accounts and then proceed with a correlation attack.

One unique concern for pathogen genome databases is that the knowledge of pathogen sequences may lead to malicious use. Such ill-intended use of genomic data and technology is a major biosecurity concern. Currently, many genomes of animal and plant pathogens are freely accessible to any user through pathogen genome databases. A study in early 2000s had concluded that open access to pathogen genomes should be promoted (Committee on Genomics Databases for Bioterrorism Threat Agents et al., 2004). However, situations have changed due to the reduced cost of synthetic DNA technology and advancement in synthetic biology (Hughes and Ellington, 2017). Even the genome sequence of such a high-risk pathogen as the smallpox virus, Variola major, can be easily accessed at NCBI by any anonymous user. Putting in place more stringent regulations regarding access to sensitive data by governments is one potential solution for this problem. However, it is challenging to determine what should be regulated and what should not be regulated, particularly in a collaborative research setting. Imposing such regulations may also discourage research groups to conduct research related to these pathogens and increase the operating costs for those groups. In our opinion, one possible model is that, instead of granting free access to pathogen genomes to anyone who has an internet connection, funding agencies could control the access to genomic information for high-risk pathogens. Genomic data for these pathogens would only be available once the corresponding grant application has been peer-reviewed and determined as fund-able.

**Data integrity**. Genome databases grow rapidly due to the increasing amount of sequencing data. Many genome databases have protocols for data quality control and manual curation, which are two methods to ensure data integrity. For all databases reviewed in this article, to submit a new data set to these databases, a user has to register an account with an email address and the data submitted to the database cannot be directly inserted in the main database. There is always a curator or an administrator to oversee the process. In several cases, a user can upload his own data to the server and perform analysis using the web interface provided by the database. However, user-provided data cannot be directly integrated into the main database in any situation. Many web sites provide methods for users to upload data. Interestingly, there seems to be no case where the data integrity is checked during the transfer process to ensure that the data provided by the user is not modified during the data transfer process. The rapid growth of the genomic and bioinformatic fields has also created a volume processing challenge for curators, where data science has introduced database sizes in the peta- and exa-byte range that has left institutions scrambling to bring massive “big data” computing infrastructures on-line and growing at a schedule that keeps pace with the growth of available data. Almost all traditional cybersecurity solutions fail at data volume, velocity, and variety of this scale.

Attackers have several options to exploit an unverified data transfer process. One possibility of attack is to provide invalid data, motivated to guide future studies toward specific outcomes. This attack requires careful crafting of records in the database to maintain a valid format but containing data without experimental evidence. This attack can be done during a single data transfer. The mitigation is to use thorough analysis of the data at transfer time. The analysis can easily ensure correct formatting of the data, discarding garbled input. However, verifying the validity of the data is particularly challenging and cannot be easily performed using existing methods. Another type of attack consists in gradually injecting invalid records within a larger valid data set. For example, the attacker could download existing data from the database, extract a subset of the data, and inject invalid input. In this case, detection mechanisms that use probabilistic analysis can fail to find the invalid records. Only records with clear violation of data integrity can be detected. Such attacks have been proposed previously in various contexts, for example (Mo et al., 2010; Cárdenas et al., 2011; Esmalifalak et al., 2013).

**Data availability**. Reduced data availability is a potential concern for genomic databases. This will cause delay in progress for time-sensitive experiments. For example, if a diagnostic lab is using DNA sequences as a method to identify pathogens, disruption of a database will cause delays in obtaining an identification. However, it is hard to estimate how many research projects or clinical operations do require real-time query of remote servers or databases. One major reason for loss of data availability is that web sites or databases are no longer maintained. In some cases, an older version of a website (NMPDR) is superseded by a new website (PATRIC). In several cases (not listed in this article), the web site link simply becomes obsolete. Another reason for loss of data is the adoption of distributed data models across shared high-performance research networks. A database may be freely shared in such a collaboration space, but there are not always resources to keep the database online and available for sharing with future users. It is hard to estimate the impact of such loss of availability. However, the manual labor for data curation, system and data integration, and web site development are lost.

To maintain data availability, a distributed network of permanent data providers is needed (Jsang et al., 2007). The network can include centralized control systems that can provide freshness guarantees and can maintain availability when some data providers are no longer responsive. The associated monetary cost, performance issues, and organizational aspects of this network require careful considerations.

**Attack on physical hardware**. In some databases that we have reviewed, MySQL query, a REST API, and a PERL API are provided for remote users to query data directly. In these scenarios, the databases are susceptible to attacks such as SQL injection. However, there is a limited public record of how many genome databases have been attacked by these means. Several databases provide computational tools to annotate microbial genomes, perform genome assembly, and search genome database. These computational analyses typically require substantial computing power. Many major research universities are equipped with cluster computing servers that have been used as the backends for these computationally intensive services. Therefore, these servers are attractive targets for malicious usage such as mining of cryptocurrency (Tahir et al., 2017).

**Future physical exploitation**. As genomic data become an integral part of an individual's healthcare and treatment plan, the traditional firewall between bioinformatics and medical technology becomes more porous. Thus, an acceptable operating risk for a genome database may be transmitted downstream, where it becomes an unacceptable threat to the technology responsible for a patient's care. As this threat scenario evolves from the hypothetical to the possible, today's low risk research data will become the foundation for the high risk and critical health care analyses; security controls that today seem to lack a Return On Investment (ROI) for their overhead costs will have to be retrofitted, or the whole body of work will have to be revalidated and secured properly.

In summary, genome databases will only grow as a target for multiple existing cybersecurity threats and threat actors. Users of genome databases could lose their personal information, such as an email address and associated password. Patients and subjects of research that capture genomic data may find that institutions have lost control of the most intimate data available about them. Many genome databases have established local standards for data quality and metadata curation and include administrators to oversee the process of data upload but have not engaged with current cybersecurity best practices and industry standards to protect the systems and networks upon which they rely. Data availability can affect users productivity but the actual costs of malicious cyber activity in the genomics field is difficult to quantify, and no one has accurately developed a methodology for financial loss estimates. Yet the lack of a measure of the risk does not negate the very real risks that exist.

## 4. Security Requirements and Potential for New Approaches

In this section, we discuss the existing security measures used by pathogen genome databases and what can be potentially improved in current practices.

**Access control**. Many databases reviewed in this article contain components that do not require login. Users can simply use a web interface to query data from databases such as NCBI, EMBL-EBI, and many other specialized databases. Users also can use a programming language, a REST API, or a MySQL query to access data. For batch download, anonymous FTP access is provided in several cases. Both, NCBI and EMBL-EBI, have implemented speed limits for bulk download using programmable interfaces. IP block is used by NCBI to limit download speed. Almost all databases require users to use email and password as methods for login to gain access to data upload and data analytic capability. We notice that most databases do not require strong passwords, such as combinations of long phrases, capital letters, symbols, and number. No databases reviewed in this article require two-factor authentication or login through third party accounts. Requiring strong passwords, implementing two-factor authentication, and implementing login through third party accounts (Google, ORCID, or institution-specific accounts) could provide additional security measures for the current generation of genomic databases.

Database access control systems is a well studied subject (Bertino et al., 1996; Kalam et al., 2003), with implications for mobile (Xu et al., 2016) and web applications (Xu et al., 2017). Classical access control systems, for example access control matrix, could be used (Sandhu, 1992) to provide basic functionality for systems that interface genomic databases. The core challenge here is to accommodate special use cases that are standard in the genomic research community, maintaining usability and performance while achieving high assurances.

**Data integrity check and protection**. Most databases allow users to contribute data and implement metadata standards. However, it is unclear how databases ensure that the data are intact during the transfer process. Simple methods such as cryptographic checksums could be implemented to ensure data integrity. There are concerns that malicious users can inject large amounts of useless data to public databases. Current quality control mechanisms do not allow the curators to control data quality with regard to the above mentioned, hypothetical situation. However, a large data set upload is controlled by the database administrator such that it is unlikely that random, large data sets can be integrated into a database without being noticed. Another possibility is that malicious users can modify certain records in a public database. However, it is difficult to imagine the motivation for performing such an attack on public genome databases. Another model for data protection is the use of encrypted databases (Eykholt et al., 2017) or the use of secure multiparty computation (Evans et al., 2018). For example, access to databases does not have to be binary, allowing or denying access based on access control models. One can reveal partial views of the database as needed.

**Data availability and longevity**. Loss of database access entails loss of valuable research results and waste of manual labor in the data curation process. Since maintenance of online databases requires continuous manual support, it is common that some databases cannot be maintained due to lack of funding support. One solution to this problem is to deposit data to public databases that are maintained by national governments such as the databases managed by NCBI. Two examples we reviewed are FDA-ARGOS and FDA-GenomeTrakr projects. Neither of these projects maintain their own databases for the data generated by these projects. Instead, data are uploaded as BioProjects to the NCBI database. This approach provides better guarantee for longer term availability of research data. NCBI BioProject and GEO databases provide a good repository for genomic data and these databases are not limited to the deposit of raw data alone.

## 5. Conclusions

In the past 30 years, pathogen genome databases and genome databases in general have become an integral part of biological and biomedical research. Although genome databases have not been reported as primary targets of cybersecurity threats, many common cybersecurity threats are applicable to genome databases. Disrupting genome databases can lead to loss of productivity, loss of research investment, and loss of private data, such as email addresses and passwords. Computing servers used by genome databases can be hijacked for cryptocurrency mining or other malicious purpose. Since the revolution of genomic science started by sequencing human genomes, billions of research funding have been invested in performing genomic experiments, generating genomic data, annotating, curating, and interpreting genomic data. Despite this large investment in genomic sciences, we found there is almost no dedicated research that focuses on protecting such data from cybersecurity threats. We think that it is necessary for the community that develops genomic databases to collectively design a minimum, necessary security standard for new genome database projects.

## Author Contributions

SL developed the first draft of the manuscript. BV, LH, HA, MS, and CL provided comments and made edits to the manuscript.

### Conflict of Interest Statement

The authors declare that the research was conducted in the absence of any commercial or financial relationships that could be construed as a potential conflict of interest.
